# *Brucella Melitensis* 16M Regulates the Effect of AIR Domain on Inflammatory Factors, Autophagy, and Apoptosis in Mouse Macrophage through the ROS Signaling Pathway

**DOI:** 10.1371/journal.pone.0167486

**Published:** 2016-12-01

**Authors:** Tiansen Li, Yafang Xu, Laizhen Liu, Meiling Huang, Zhen Wang, Zhixia Tong, Hui Zhang, Fei Guo, Chuangfu Chen

**Affiliations:** 1 College of Animal Science and Technology, Shihezi University, Shihezi, Xinjiang, China; 2 College of life science, Shihezi University, Shihezi, Xinjiang, China; 3 College of medicine, Shihezi University, Shihezi, Xinjiang, China; University of Pittsburgh, UNITED STATES

## Abstract

Brucellosis is a highly contagious zoonosis caused by *Brucella*. *Brucella* can invade and persist inside host cells, which results in chronic infection. We constructed AIR interference and overexpression lentiviruses to acquire AIR interference, overexpression, and rescue stable expression cell lines. We also established a *Brucella melitensis* 16M-infected macrophage model, which was treated with either the vehicle control or NAC (ROS scavenger N-acetylcysteine (NAC) for 0, 3, 6, 12, and 24 h. Confocal laser microscopy, transmission electron microscopy, fluorescence quantitative PCR, flow cytometry, ELISA, and Western blot were used to detect inflammation, cell autophagy and apoptosis-related protein expression levels, ROS levels, and the distribution of mitochondria. It was found that after interference and overexpression of AIR, ROS release was significantly changed, and mitochondria became abnormally aggregated. *B*. *melitensis* 16M activated the NLRP3/AIM2 inflammatory complex, and induced RAW264.7 cells to secrete IL-1β and IL-18 through the ROS pathway. *B*. *melitensis* 16M also altered autophagy-related gene expression, increased autophagy activity, and induced cell apoptosis through the ROS pathway. The results showed that after *B*. *melitensis* 16M infection, ROS induced apoptosis, inflammation, and autophagy while AIR inhibited autophagosome maturation and autophagy initiation. Autophagy negatively regulated the activation of inflammasomes and prevented inflammation from occurring. In addition, mitophagy could promote cell apoptosis.

## Introduction

*Brucella* is a facultative intracellular parasite that invades and persists inside host macrophages[1]. Macrophages, as important immune cells in the body, play an important role in removing and controlling *Brucella*[2]. Macrophages can kill most of the invading *Brucella*, but a small portion is able to evade the immune system and survive and propagate in macrophages[3]. In addition, *Brucella* can induce macrophage apoptosis, and thus, evade the immune system[4]. Worse still, *Brucella* spread apoptotic macrophages throughout the body, causing chronic infection[5]. *Brucella* can also survive in infected hosts by regulating monocyte apoptosis[6]. Unfortunately, the specific intracellular survival mechanisms and immune escape mechanism are not yet clear.

*Brucella* infection can cause damage to joints and the nervous, reproductive, and immune systems[7]. The symptoms of brucellosis in animals include abortion, orchitis, and arthritis[8]. *Brucella* is similar to Bennett Rickettsia in that *Brucella*-containing vacuole (BCV) also follows the endocytic-lysosomal pathway [9]. In addition, the type IV secretion system of *Brucella* is involved in the regulation of this process[10]. In the late stage of the cycle, the replicative BCV (rBCV) derived from endoplasmic reticulum is converted into autophagic BCV (aBCV)[11]. This process requires the autophagy initiation protein Ulk-1, autophagy gene *Beclin 1*, and *Atg14L*[12]; this shows that atypical and classical autophagy induced by *Brucella* share common upstream regulatory pathways[13]. Smooth *Brucella* inhibits macrophage apoptosis, while rough *Brucella* induces macrophage apoptosis[5]. In *Brucella*-infected monocytes, the *Bcl-2* family member *A1* gene, which is associated with blood cell survival, is overexpressed[14]. Infected macrophage-like cells can inhibit the Fas-ligand or γ-interferon-induced apoptosis, indicating that the immune system adapts its response against the cell toxicity caused by an infection so that the infected host cells can be protected[15].

Tectonin β-propeller repeat containing 1 (TECPR1) is characterized as a protein that functions in the autophagosome maturation process by promoting the fusion of autophagosomes with lysosomes and it also plays a role in selective autophagy in the innate immune response[16]. The Atg12–Atg5-interacting region (AIR) domain in the membrane fusion-associated protein TECPR1 plays an important role in the process of autophagy[17]. The AIR binds to the Pleckstrin homology (PH) domain and induces autoinhibition to block the interaction between the PH domain and phosphatidylinositol 3-phosphate (PtdIns3P) molecules of the autophagosomal membrane and thereby regulates the autophagic function of TECPR1[17]. TECPR1 belongs to the TECPR1 family, which is a selective autophagy membrane fusion-associated protein. TECPR1, containing AIR and PH domains, interacts with Atg5, and thus, plays a role in autophagy[18, 19]. Atg5 can bind to the ubiquitin-like protein Atg12, and plays a vital role in autophagy[20]. Studies have shown that during the selective autophagy process, TECPR1 exerts its function by targeting pathogens, such as damaged mitochondria and protein aggregates[16]. TECPR1 is also critical for the maturation of autophagosomes as well as promoting the fusion of autophagy and lysosome[21]. AIR, an important domain of TECPR1, can bind to the autophagy-associated protein complex Atg12-Atg5, and liberates the PH domain from Atg12-Atg5 binding, thus promoting the fusion of autophagosomes and lysosomes[19].

Reactive Oxygen Species (ROS) is the second messenger of cellular apoptosis[22]. After cells receive pro-apoptotic signals, ROS production is increased, which may lead to increased Ca^2+^ influx, upregulation of Bax, opening of the MPTP, caspase activation, and eventually cell death[23]. The anti-apoptotic protein Bcl-2 can inhibit ROS-induced lipid peroxidation by suppressing ROS production[24]. Studies have confirmed that *Brucella* infection can cause autophagy, inflammation, and apoptosis[25]; however, whether it is through the AIR domain or the ROS pathway has not been reported. Therefore, this study systematically explored whether the inflammation, autophagy, and apoptosis caused by *Brucella* infection is associated with the AIR domain and ROS pathway. This evaluation laid the foundation for the future study of immune evasion, intracellular survival of *Brucella*, and drug and vaccine development against *Brucella* infection.

## Materials and Methods

### Bacterial Strains and Growth Conditions

*Brucella melitensis* 16M (Chinese Center for Disease Control and Prevention; Beijing, China) was cultured in tryptic soy agar (TSA) or tryptic soy broth (TSB) medium (Oxoid, UK) in either a static 37°C incubator or a 37°C shaker. *E*. *coli* strain DH5α (Chinese Center for Disease Control and Prevention; Beijing, China) was cultured with Luria-Bertani (LB) culture medium containing antibiotics (50 mg/ml of ampicillin) (Table 1).

**Table 1:** Bacterial Strains and Cells.

Bacterial Strains and Cells	Plasmids	Infected Cells	Source
*Brucella melitensis* 16M			Center for Disease Control and Prevention
*E*.*coli* DH5α			
Human embryonic kidney cells HEK-293FT			Shanghai Institute of Cell Biology
Mouse macrophages RAW264.7			
I-A group	pLL3.7-AIR	RAW264.7	In this study
O-A group	pLEX-AIR	RAW264.7	In this study
OA-IA group	pLEX-AIR	I-A	In this study
RAW264.7-LC3	pcDNA3.1-GFP-LC3	RAW264.7	In this study
I-A-LC3 group	pLL3.7-AIR	RAW264.7-LC3	In this study
O-A-LC3 group	pLEX-AIR	RAW264.7-LC3	In this study
OA-IA-LC3 group	pLEX-AIR	I-A-LC3	In this study

### Cells and Growth Conditions

HEK-293FT cells and mouse macrophages RAW264.7 (Shanghai Institute of Cell Biology, Chinese Academy of Sciences, Shanghai, China) (Table 1), were cultured in DMEM medium containing 10% FBS (Gibco, USA) and set in a 37°C, 5% CO_2_ incubator.

### Plasmids

Lentiviral overexpression vector pLEX-AIR was constructed by our lab. pLEX-MCS, pLEX-EGFP, and enveloping and packaging vectors (pSPAX2 and pMD2.G) were purchased from Xinjiang Academy of Animal Science (Xinjiang, China). Lentiviral RNAi vector pLL3.7- AIR was constructed by our lab. pLL3.7-EGFP and helper plasmids PRSV-REV and PCMV-VSVG were purchased from Xinjiang Academy of Animal Science (Xinjiang, China). pMD19-T cloning vector was purchased from Takara (Dalian, China).

### AIR Interference Vector and Overexpression Vector and Identification of Stable Expression Cell Lines

According to the GenBank sequence of the mouse *TECPR1* (Accession No. NM_027410), we designed PCR amplification primers (Table 2) for the *AIR* gene using Premier 5.0 software (Premier, Canada) while qRT-PCR primers for the mouse *AIR* gene (Accession No: NM_027410.1) and *β-actin* (Accession No: EF095208. 1), were designed according to the GenBank sequences (Table 2). Total RNA was extracted (RNA extraction kit, Cwbiotech, China) from cultured murine macrophages RAW264.7, and cDNA was synthesized from RNA through reverse transcription (HiFi-Script cDNA first strand synthesis kit, Cwbiotech, China). The *AIR* gene was amplified using *AIR*-F and *AIR*-R primers, and was ligated to the pMD19-T vector, which was digested by *BamH* I and *Xho* I and ligated to pLEX-MCS to generate pLEX-AIR. We also constructed pLL3.7-AIR using the similar subcloning strategy. HEK-293FT and RAW264.7 cells were transfected with the lentiviral packaging plasmids pLEX-AIR and pLL3.7-AIR, with the ratio of 5 ml of plasmids for 2 × 10^5^ cells. Plasmids and cells were thoroughly mixed and placed in a 37°C, 5% CO_2_ incubator. AIR stable cell interference (I-A group), AIR stably overexpressing cells (O-A group), and AIR overexpression-inference cells (OA-IA group) were generated. Stable cell lines were observed by fluorescence microscope (Nikon, Japan) 48 h post-transfection. RAW264.7-LC3, I-A-LC3, O-A-LC3, and OA-IA-LC3 cells were generated using the same method.

**Table 2:** Primers for amplifying and real-time quantitative PCR.

Genes	Primers	Primer sequences(5'→3')
AIR	qAIR-F	GTCCCTGTCCATCACCCC
	qAIR-R	TCACCCACACTGACTGCTCC
β-actin	β-actin-F	AGCCTTCCTTCTTGGGTATGG
	β-actin-R	CCTGTCAGCAATGCCTGGGTA
NLRP3	NLRP3-F	TGGAGACACAGGACTCAGGC
	NLRP3-R	CATTTCACCCAACTGTAGGC
AIM2	AIM2-F	CTAACCACGAAGTCCCAAAT
	AIM2-R	TTCCCAGCACCAACACC
ASC	ASC-F	CTGGTCCACAAAGTGTCCTG
	ASC-R	GCAACTGCGAGAAGGCTAT
Caspase-1	Caspase-1-F	TGCCGTGGAGAGAAACAA
	Caspase-1-R	ATGAAAAGTGAGCCCCTG
L7/L12	L7/L12-F	ATGGCTCATCTCGCAAAGA
	L7/L12-R	TTACTTGAGTTCAACCTTG
p62	p62-F	TCTTTGGACCCCCGTGTGA
	p62-R	TCTCACAGATACCCCACGACCA
LC3-I	LC3-I-F	CCGACCGCTGTAAGGAGG
	LC3-I-R	GCCGGATGATCTTGACCAAC
LC3-II	LC3-II-F	GAACAAAGAGTGGAAGATG
	LC3-II-R	GCCGTCTGATTATCTTGA
Bax	Bax-F	GCCTTTTTGCTACAGGGTTT
	Bax-R	TGCTGTCCAGTTCATCTCCA
Bcl-2	Bcl-2-F	GACTTCTCTCGTCGCTACCG
	Bcl-2-R	ACAATCCTCCCCCAGTTCAC
AIR	AIR-F	*(BamH* I) GGATCCATGCTGTCCCTGTCCATCACC*
	AIR-R	(*Xho* I) CTCGAGCACCCACACTGACTGCTCCAC*

*Underlined portions indicate the restricted sites.

### Establishment of a Cell Model of *B*. *melitensis* 16M Infection

In order to establish a cell model of *B*. *melitensis* 16M infection, PBS and NAC pretreated cells were infected with *B*. *melitensis* 16M at the logarithmic growth phase. MOI (bacteria: cells) was 100:1. All cells were cultured in DMEM medium containing 10% FBS, at 37°C, in a 5% CO_2_ incubator. After 1 h, gentamycin (30 μg/ml, Sigma-Aldrich) was added into the cell medium for 30 min to kill all *B*. *melitensis* 16M outside of the cells. Cells received different treatments at 0, 3, 6, 12, and 24 h.

### qRT-PCR Detection

According to the *B*. *melitensis* 16M infection models, at 0, 3, 6, 12, and 24 h after infection, the total RNA was extracted and reverse transcribed to cDNA, with β-actin as a reference gene. Real-time PCR was conducted on the Light-Cycler 480 (Roche Applied Science, Switzerland) with the SYBR Premix Ex Taq^™^ reagent kit (TaKaRa, Dalian, China) using 2^-ΔΔCt^ analysis to calculate relative gene expression.

### Confocal Microscopy

At 0, 3, 6, 12, and 24 h after infection, DCFH-DA probes (Yeasen, USA) diluted 1,000 times with DMEM (DMEM:DCFH-DA = 1000:1) were added into the cells and placed in a 37°C, 5% CO_2_ incubator for 20 min. The cells were washed with DMEM 3 times to completely remove the DCFH-DA probe that did not enter the cells. The cells were washed another 3 times with PBS, followed by 4% paraformaldehyde (PFA) solution incubation for 30 min. After the PFA was removed, 600 μl of PBS was added to the cells and the cells were observed by a confocal microscope (ZISS, Germany). For mitochondria staining, Mito-ID^®^ Red dye (Enzo, Switzerland) was added to the cells for 25 min and incubated at 37°C. Cells were then washed with the PBS 3 times, followed by 4% paraformaldehyde (PFA) solution incubation for 30 min. After PFA was discarded, 600 μl of PBS was added to the cells and the cells were observed by confocal microscope. For apoptosis analysis, 6 h after *B*. *melitensis* 16M infection, the cells were washed with sterile PBS 3 times, and incubated with a mix of 500 μl of binding buffer, 5 μl of Annexin V-FITC, and propidium iodide for 5 min at room temperature away from light. Cells were observed under laser confocal microscopy.

### Transmission Electron Microscopy

At 6 and 12 h post-infection, 4% glutaraldehyde was added in the cells. Cells were fixed overnight at 4°C. Glutaraldehyde was removed and replaced with 1% osmium tetroxide at room temperature for 1 h. Cells were dehydrated with a gradient concentration of ethanol and infiltrated with dehydrating agent and epoxy resin (ratio of 3:1, 1:1, and 1:3, 1 h for each step). Samples were embedded and sectioned with a microtome followed by double-staining with uranyl acetate and lead citrate. Finally, samples were observed under transmission electron microscopy (JEOL, Japan).

### Cytokines

At 0, 3, 6, 12, and 24 h post-infection, cell culture supernatant was collected, and filtered through a 0.22-μm membrane filter. After centrifugation at 3,000 rpm for 10 min, cytokine levels in the supernatant, including caspase-1, IL-1β, and IL-18, were detected by ELISA (R & D, USA) as previously described[26].

### Flow Cytometry

At 3, 6, 12, and 24 h post-infection, cells were digested with EDTA-free trypsin and washed with PBS containing 2% BSA 2 times, centrifuged at 800 rpm for 5 min and resuspended with 500 μl of binding buffer mixed with 5 μl of Annexin V-FITC and 5 μl of Propidium Iodide. After incubation in the dark at room temperature for 15 min, an additional 2 ml of PBS was added into the cell suspension and detected by flow cytometry (Life Technology, USA).

### Western Blotting

Protein lysate samples were separated by 12% SDS-PAGE gel and transferred to the NC membrane for 40 min (100 mM of Tris-HCl, 150 Mm of NaCl, 0.05% Tween 20, and pH 7.2). The NC membrane was blotted with 5% non-fat milk in TBST for 1 h, followed by incubation with a primary antibody diluted with TBST for 2 h at 37°C. The membrane was washed with TBST 3 times and incubated with a secondary antibody at 37°C for 2 h. After washing with PBS 3 times, the NC membrane was stained using a HRP-DAB kit (Zhongshan Golden Bridge, Beijing, China).

The primary antibodies that we used are: mouse anti-p62 monoclonal antibody (Abcam, UK), rabbit anti-LC3A/B monoclonal antibody, rabbit anti-NLRP3 monoclonal antibody, rabbit anti-Caspase-3 monoclonal antibody (Cell Signaling Technology, USA), rabbit anti-caspase-1 p10 monoclonal antibody, and rabbit anti-β-actin monoclonal antibody (Jackson ImmunoResearch, USA). Secondary antibodies were Cy3-conjugated donkey anti-rabbit monoclonal antibody (Jackson ImmunoResearch, USA), HRP-conjugated goat anti-rabbit IgG, and HRP-conjugated goat anti-mouse IgG (Bioworld, USA).

### Statistical Analysis

SPSS Statistics 17.0 software (IBM, USA) was used for analysis. All experimental data were denoted as mean ± standard deviation ($\bar{x}$ ± SD). Independent sample *t* test was used for comparison within the group. One-way ANOVA was used for comparison between different groups. All tests were repeated 3 times.

## Results

### Lentiviral Packaging and Infection

pLL3.7 interference lentiviral vector and pLEX-EGFP lentiviral vector contained EGFP genes in the plasmid. At 48 h postinfection, cells were observed with green fluorescence. The results showed that AIR had been successfully transfected into RAW264.7 cells (S1 Fig).

### AIR Gene Expression Levels in Differently Treated Cells

**Fig 1:**
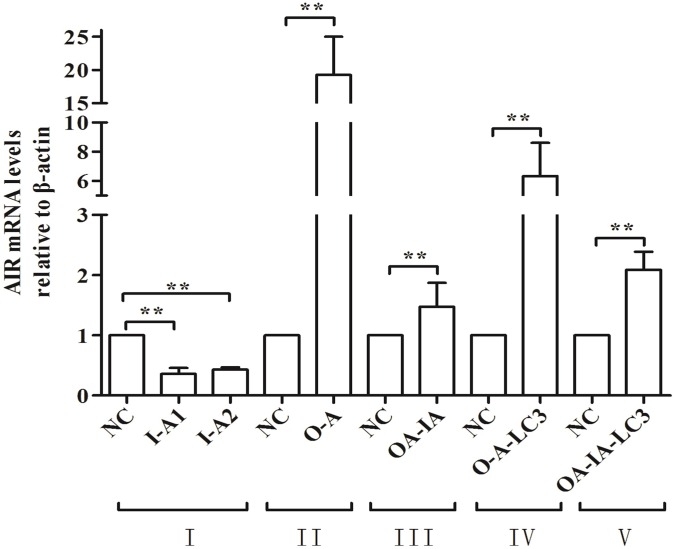
Relative expression of AIR at the mRNA level. At 48 h after infection, RAW264.7 cells infected with the AIR lentivirus vectors were completely lysed. Lysates were left at room temperature for 5 min so that the protein was completely isolated from the nucleic acid complex, and then the cells were collected. Total RNA was extracted and the concentration and purity was measured. A total of 2 μg of RNA was quantified and reverse transcribed into cDNA, which was used as the template for qRT-PCR testing.

Relative gene expression of AIR was calculated using the 2^-ΔΔCt^ method (Table 3 and Fig 1). Compared with the control group (NC), in the interference group I-A, AIR expression in cell I-A-1 and I-A-2 was significantly decreased (p < 0.01). The highest interfering efficiency reached 65%. In the overexpression O-A group and rescue OA-IA group, AIR expression in O-A-LC3 cells and OA-IA-LC3 cells was significantly increased after a lentiviral pLEX-AIR infection (p < 0.01). The results showed that we successfully constructed AIR interference, overexpression, and rescue cell lines.

**Table 3:** Detection of AIR mRNA levels in the cells of each group.

Groups		Cells	Lentivirus	Infection Cells	*Expression of AIR* gene (n = 3, $\bar{x}$ ± SD)	Inhibition/Increase rate
I	I-A	I-A-1	——	I-A-1	0.36 ± 0.10**	−64%
		I-A-2	——	I-A-2	0.43 ± 0.04**	−57%
		NC	——	RAW264.7	1.00	——
II	O-A	O-A	pLEX-AIR	RAW264.7	19.24 ± 5.77**	+1824%
		NC	pLEX-MCS	RAW264.7	1.00	——
III	OA-IA	OA-IA	pLEX-AIR	I-A-1	1.47 ± 0.40**	+47%
		NC	pLEX-MCS	I-A-1	1.00	——
IV	O-A-LC3	O-A-LC3	pLEX-AIR	RAW264.7-LC3	6.33 ± 2.28**	+533%
		NC	pLEX-MCS	RAW264.7-LC3	1.00	——
V	OA-IA-LC3	OA-IA-LC3	pLEX-AIR	RAW264.7-LC3	2.09 ± 0.30**	+109%
		NC	pLEX-MCS	RAW264.7-LC3	1.00	——

Mark: - The expression is inhibited; + The expression is increased.

Note: Compared with the control group,* indicates a significant difference (*P* < 0.05) and ** indicates an extremely significant difference (*P* < 0.01).

### ROS Levels in Differently Treated Cells Infected with *B*. *melitensis* 16M

**Fig 2:**
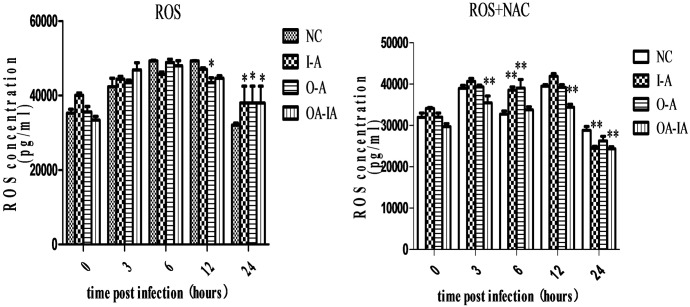
ROS production after *Brucella melitensis* 16M infection. The interference groups of I-A cells, overexpression group of O-A cells, overexpression-interference group of OA-IA cells, and the normal group of RAW264.7 cells were seeded in a 24-well cell culture plate. At 0, 3, 6, 12, and 24 h after infection, the DCFH-DA probe was added into the cells and placed in a 37°C incubator for 20 min. The cells were washed with serum-free medium 3 times to completely remove the DCFH-DA probe that did not enter the cells. We then added 600 μl of sterile PBS to the cells. ROS production was measured by using BioTek Synergy 2 Duo microplate reader.

*Brucella*-induced ROS production in RAW264.7 cells in a time-dependent manner. AIR inhibited the production of ROS, while overexpression or rescue of AIR promoted ROS production (Fig 2a). After NAC pretreatment, ROS production was significantly decreased (P < 0.01) (Fig 2b). After *B*. *melitensis* 16M infection for 6 and 12 h, ROS production in the cells was detected with confocal laser microscopy. ROS production in noninfected control cells was significantly lower than in the experimental group, and ROS production in O-A and I-A was significantly higher than the control cells while ROS production in the OA-IA group had no significant change. NAC-pretreated cells exhibited lower ROS production than the experimental group (S2 Fig).

### Distribution of Mitochondria in Different Cell Groups Infected with *B*. *melitensis* 16M

**Fig 3:**
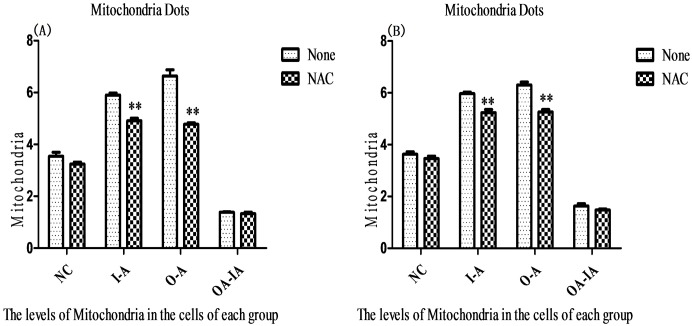
Mitochondrial distribution after *Brucella melitensis* 16M infection. At 6 (A) and 12 h (B) after infection in the interference group of I-A cells, overexpression group of O-A cells, overexpression-interference group of OA-IA cells, and the normal group of RAW264.7 cells, MIito-ID^®^ Red was added to stain the cells for 15–30 min (37°C, 25 min). After staining, the cells were washed with PBS 3 times, followed by 4% paraformaldehyde solution incubation for 30 min. After PFA was removed, 600 μL of PBS was added.

At 6 and 12 h after *B*. *melitensis* 16M infection, a confocal laser microscope was used to detect the distribution of mitochondria (labeled with Mito-ID^®^RED) in the cells. I-A and O-A cells showed an abnormally significant higher accumulation of mitochondria compared with the control cells, negative control cells, and OA-IA cells (P < 0.01). The results indicated that ROS could cause cell damage by activating the inflammasomes. Therefore, mitochondria-derived ROS could activate the NLRP3 inflammasomes. After NAC pretreatment, abnormal aggregation of mitochondria in I-A and O-A cells was significantly higher than in the control cells, negative control cells, and OA-IA cells, but lower than in the untreated cells (P < 0.01) (S3 Fig and Fig 3). TEM images showed that after *B*. *melitensis* 16M infection for 6 hours and 12 hours, I-A, O-A, and OA-IA cells showed significant abnormal aggregation of mitochondria. Mitochondria were enlarged and became round shaped and mitochondria showed multifocal vacuolar degeneration, with a lighter matrix and less cristae. The cristae structure became less clear and more disorganized, and some showed multiple cystic crests. The center of the mitochondria showed small particles or large vacuoles, indicative of damaged mitochondria. After NAC pretreatment, mitochondria showed similar aggregation and damage, but to a lesser degree compared with the untreated cells (S4 Fig).

### Caspase-1, NLRP3, AIM2, and ASC Gene Expression Levels in *B*. *melitensis* 16M-Treated Cells

**Fig 4:**
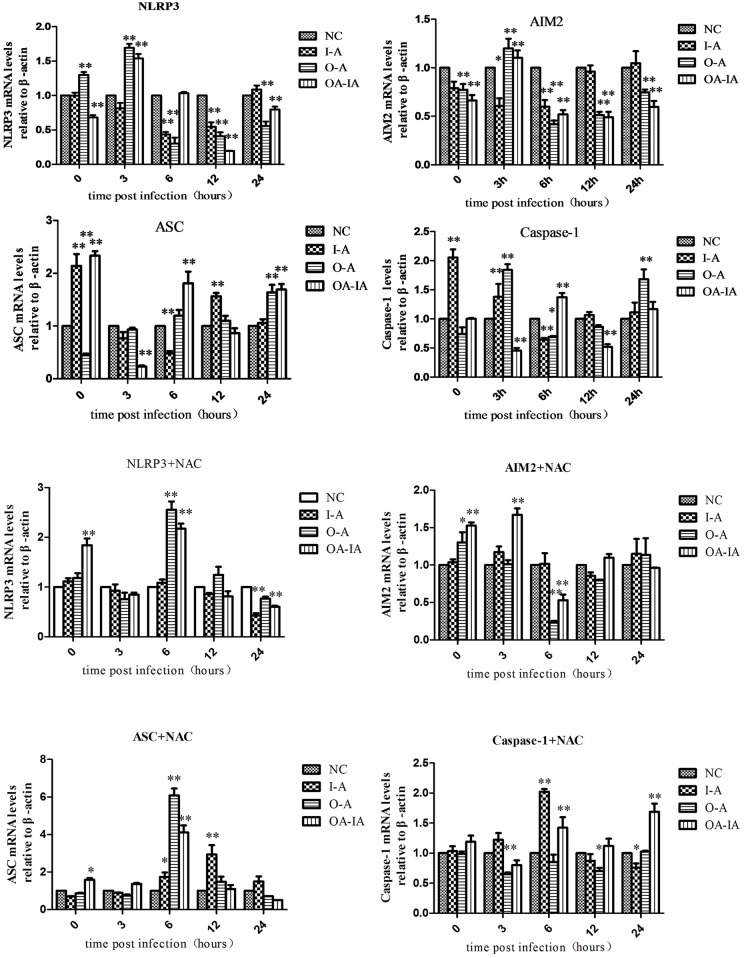
Expression of inflammatory factors in differently treated cells were detected by ELISA. Both the untreated and NAC-pretreated groups were infected with *Brucella melitensis* 16M. At 0, 3, 6, 12, and 24 h after infection, the total RNA was extracted and reverse transcribed into cDNA, which was quantitated by qRT-PCR.

Relative expression of Caspase-1, NLRP3, AIM2, and ASC levels in the transcript level was calculated using the 2^-ΔΔCt^ method (Fig 4).

NLRP3 protein exhibited an apparent change at 6 and 24 h after *B*. *melitensis* 16M infection, while the AIM2 protein underwent a marked change at 6 h. At 6 h after *B*. *melitensis* 16M infection, NLRP3 and AIM2 in the O-A and OA-IA cells were significantly higher than in the control cells (P < 0.01), indicating that at 6 h *B*. *melitensis* 16M infection activated NLRP3 and the AIM2 inflammasome. At 24 h after *B*. *melitensis* 16M infection, NLRP3 and AIM2 in the I-A, O-A, and OA-IA cells were lower than in the control group and NLRP3 and AIM2 in the I-A cells were significantly different from those in the OA-IA cells (P < 0.01), suggesting *B*. *melitensis* 16M infection inhibited NLRP3 and AIM2 inflammasome activation. Adapter protein ASC and Caspase-1 followed the same trend with NLRP3 and AIM2 inflammasomes, indicating that Caspase-1 could activate NLRP3 and AIM2 inflammasomes and thereby caused inflammation. The change of Caspase-1 protein level was consistent with the change of ASC, which suggested that *Brucella* infection, similar with other intracellular parasites, activated NLRP3 inflammasomes and shares the same molecular mechanisms with a variety of other pathogenic microorganisms, especially facultative intracellular parasites.

### ELISA Detects IL-1β, IL-18, and Caspase-1 Levels in *B*. *melitensis* 16M-Infected Cells

**Fig 5:**
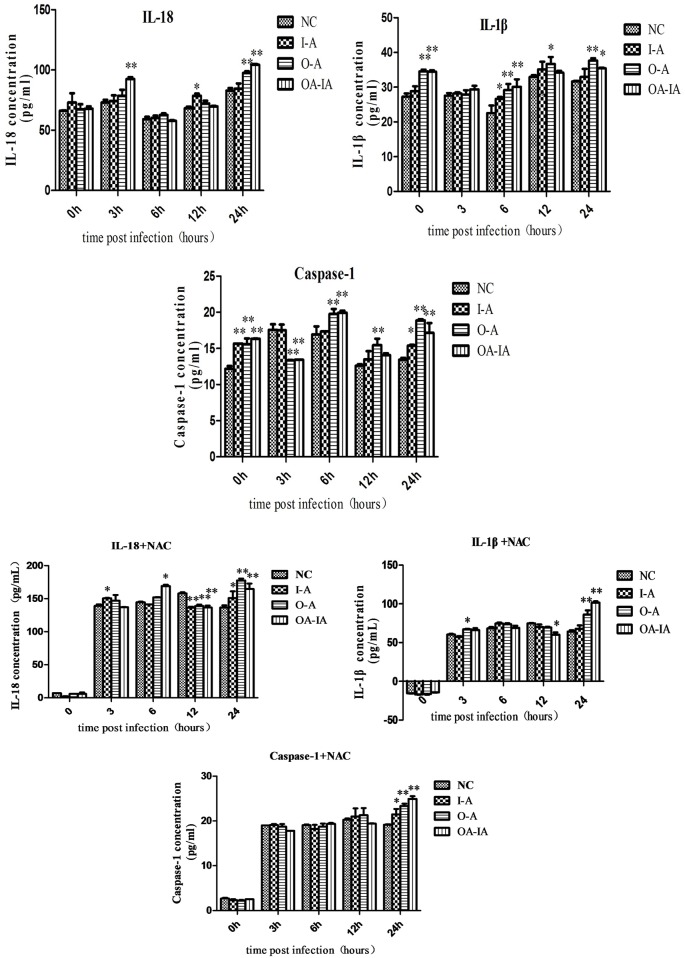
IL-1β, IL-18, and Caspase-1 expression were detected by ELISA. Both the untreated and NAC-pretreated groups were infected with *Brucella melitensis* 16M. At 0, 3, 6, 12, and 24 h after *Brucella melitensis* 16M infection, the cell supernatants were collected and filtered through a 0.22-μm membrane. After centrifugation at 3,000 rpm for 10 min, the supernatant was transferred to a new EP tube. Inflammatory factors were examined by ELISA.

Compared with the control group, infection with *B*. *melitensis* 16M cells led to a change of the inflammatory cytokines IL-1β and IL-18 to varying degrees (Fig 5). *B*. *melitensis* 16M infection induced the maturation and secretion of downstream proinflammatory cytokines. Secretion of IL-lβ depends on the activation of inflammatory complexes. Different pathogens activate different inflammatory complexes and induce the secretion of IL-lβ so no single molecule was involved in *Brucella*-induced IL-lβ and IL-18 secretion, but rather a variety of inflammatory complexes were involved in the regulation of IL-lβ and IL-18 during different infectious periods. Results from the NAC-pretreated group showed that NAC could cause Caspase-1 activation, resulting in the increased secretion of inflammatory cytokines, and thereby activating the NLRP3 and AIM2 inflammasomes (Fig 5).

### NLRP3 and Caspase-1 p10 Protein Expression Levels in *B*. *melitensis* 16M-Infected Cells

After *B*. *melitensis* 16M infection in differently treated cells, Western blot was used to detect the expression of NLRP3 protein levels at all of the time points (S5 Fig). NLRP3 protein expression in the differently treated groups was all lower than in the control group. Pretreatment with NAC upregulated the expression of NLRP3, which indicated that ROS activated the NLRP3 inflammasome.

After *B*. *melitensis* 16M infection in differently treated cells, Western blot was used to detect the expression of Caspase-1 p10 protein levels at all of the time points (S5 Fig). Caspase-1 p10 protein expression in the experimental groups was all lower than in the control group. Conversely, NAC pretreatment upregulated the Caspase-1 p10 protein level, suggesting that NAC activated Caspase-1 and thereby increased the secretion of inflammatory cytokines.

### Co-localization of LC3 and Mitochondria in *B*. *melitensis* 16M-Infected Cells

**Fig 6:**
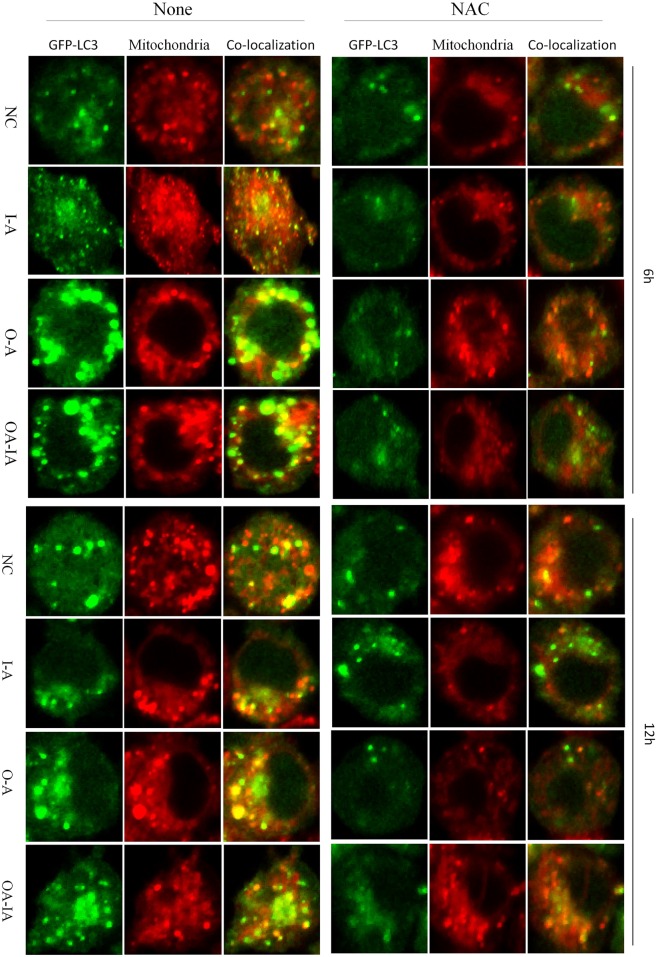
MIito-ID^®^ Red probe was used to detect the distribution of mitochondria induced by *Brucella melitensis* 16M. The interference groups of I-A-LC3 cells, overexpression group of O-A-LC3 cells, overexpression-interference group of OA-IA-LC3 cells, and the normal group of RAW264.7 cells were passaged and seeded into confocal dishes. Both untreated and NAC-pretreated groups were set up and infected by *Brucella melitensis* 16M. At 6 and 12 h after infection, mitochondria distribution and GFP-LC3 expression were examined under a confocal laser microscope.

After *B*. *melitensis* 16M infection, GFP-LC3 was dispersed in the control cells while experimental groups showed punctate aggregates (Fig 6), which indicated cell autophagy upon the infection of *B*. *melitensis* 16M. After AIR interference, GFP-LC3 punctate aggregates became more significant (Fig 6), suggesting that interference of AIR promoted autophagy. NAC pretreatment decreased intracellular GFP-LC3 punctate aggregates (Fig 6), indicating that autophagy was inhibited. I-A and O-A cells showed less abnormal mitochondrial aggregates than the control cells (Fig 6), suggesting that NAC could reduce the mitochondrial damage caused by *B*. *melitensis* 16M.

### Autophagosome Formation in *B*. *melitensis* 16M-Infected Cells

A transmission electron microscope was used to observe the formation and distribution of cell autophagosomes 6 h after *B*. *melitensis* 16M infection (S6 Fig). In I-A and O-A cells, there were a large number of vacuoles with a bilayer membrane structure, namely autophagic vacuoles, suggesting more cell autophagy in the I-A and O-A groups, while the control and OA-IA groups showed less autophagic vacuoles. NAC pretreatment reduced the number of autophagic bodies, indicating that ROS could promote autophagy.

### Expression Level of Autophagy-Associated Genes in *B*. *melitensis* 16M-Infected Cells

Relative expression levels of LC3A, LC3B, and p62 were calculated using the 2^-ΔΔCt^ method.

**Fig 7:**
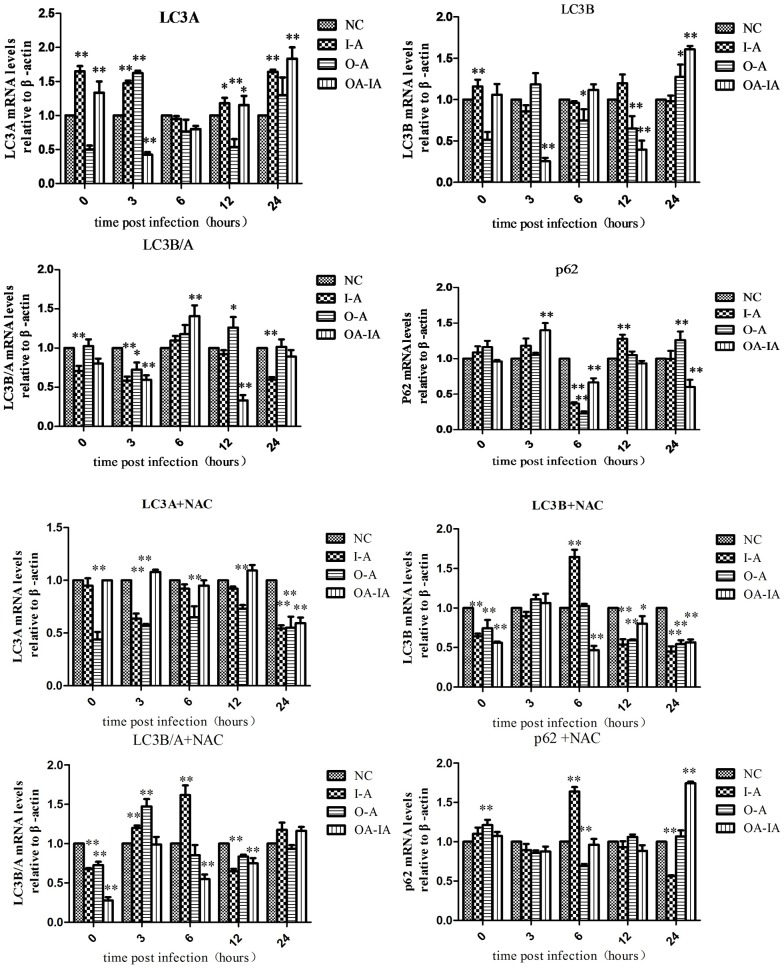
mRNA level of autophagy-associated gene expression by qRT-PCR. Both the untreated and NAC-pretreated groups were setup and infected with *Brucella melitensis* 16M. At 0, 3, 6, 12, and 24 h after infection, the total RNA was extracted and reverse transcribed into cDNA, which was subjected to qRT-PCR detection.

Cell autophagy-related gene expression levels in *B*. *melitensis* 16M-infected but non-NAC-treated cells are shown in Fig 7. Compared with the control group, LC3A in the interference group reached the highest at 6 h; LC3B was first reduced and then gradually increased and peaked at 6 h. Compared with the control group, P62 expression in the interference and overexpression groups was suppressed at 6 h, but was increased at other time points, indicating that autophagy was promoted at 6 h but was inhibited otherwise. After NAC pretreatment (Fig 7), compared to the negative control group, LC3A levels in the interference and overexpression groups were decreased, but the difference was not significant. LC3B in the interference group was gradually increased and peaked at 6 h, and the difference was significant (p < 0.01); however, the LC3B protein level dropped afterwards. P62 in the interference group reached the highest point at 6 h, and the difference was significant (p < 0.05), suggesting that NAC inhibited autophagy.

### P62 Protein Levels in *B*. *melitensis* 16M-Treated Cells

Western blot showed the expression level of p62 at different time periods after *B*. *melitensis* 16M infection (S7 Fig). At 6 h, p62 protein expression in the I-A group was significantly lower than in the control group. It showed that after the AIR gene was silenced, compared with the control group, the relative expression level of the p62 protein showed a significant change, indicating that after AIR was silenced, autophagy activity was increased. After NAC pretreatment, at 6 h, p62 expression in the I-A group was increased, suggesting that NAC pretreatment of I-A cells inhibited autophagy activity. Compared with the control group, the overexpression and rescue groups showed no significant change in p62 protein levels.

### Expression Levels of Apoptosis-Associated Genes in *B*. *melitensis* 16M-Infected Cells

Relative expression of Bcl-2 and Bax in the transcription levels were calculated using the 2^-ΔΔCt^ method.

**Fig 8:**
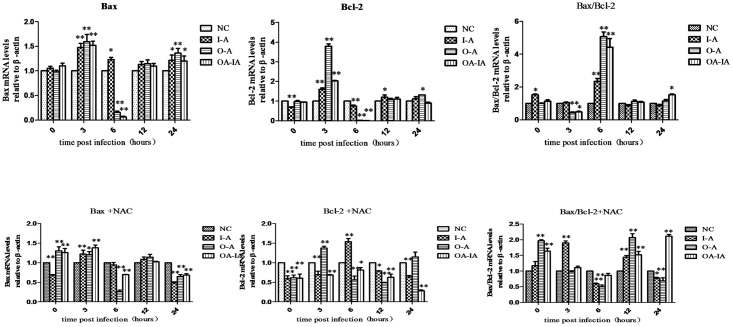
mRNA level of apoptosis-associated gene expression by qRT-PCR. Both the untreated and NAC-pretreated groups were set up and infected with *Brucella melitensis* 16M. At 0, 3, 6, 12, and 24 h after infection, the total RNA was extracted and reverse transcribed into cDNA and quantitated by qRT-PCR.

Expression levels of the apoptosis-associated genes in the *B*. *melitensis* 16M-infected but NAC-untreated cells are shown in Fig 8. At 6 h, the ratio of Bax/Bcl-2 in the I-A, O-A, and OA-IA groups was significantly higher than in the control group (P < 0.01).

Expression levels of apoptosis-associated genes in the *B*. *melitensis* 16M-infected and NAC-treated cells are shown in Fig 8. At 6 and 24 h, the ratio of Bax/Bcl-2 in the experimental groups was significantly lower than in the control group (P < 0.05) or (P < 0.01). At 12 h, the ratio of Bax/Bcl-2 in the *B*. *melitensis* 16M-infected experimental groups was significantly higher than that in the control group (P < 0.01).

### Apoptosis in *B*. *melitensis* 16M-Infected Cells

**Fig 9:**
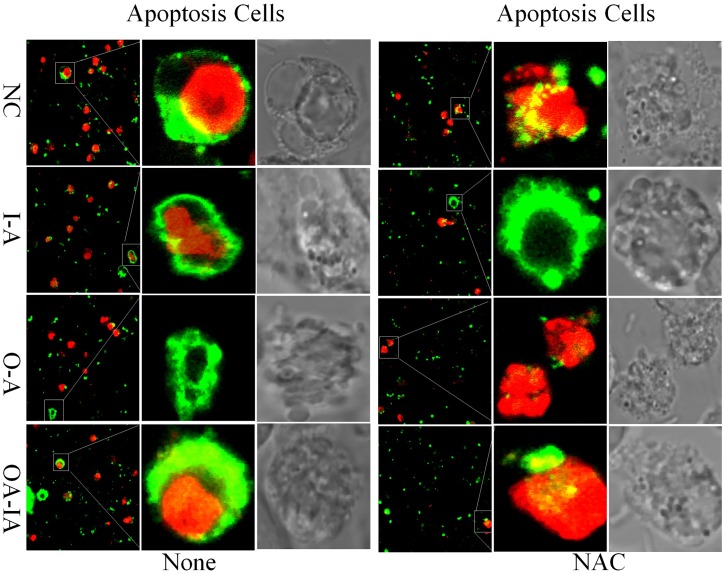
Confocal laser microscopy to detect cell apoptosis in different treatment groups. Cells were seeded into a confocal dish and infected with *Brucella melitensis* 16M at MOI (50:1). At 6 h after infection, the cells were washed with PBS 2–3 times, and incubated with a mix of 500 μL of binding buffer, 5 μL of Annexin V-FITC, and Propidium Iodide at an ambient temperature away from light for 5 min. Cell apoptosis was observed under confocal microscopy.

At 6 h after *B*. *melitensis* 16M infection, confocal laser microscopy images of apoptotic cells are shown in Fig 9. Compared with the control group, after *B*. *melitensis* 16M infection, the green- and red-stained cells increased. After NAC pretreatment, compared with the control group, *B*. *melitensis* 16M infection resulted in fewer green- and red-stained cells. Phase contrast images showed the occurrence of cell apoptosis and marked the changes in cell morphology. Apoptotic cells were separated from surrounding cells and exhibited a smaller cell size, deformation, and cell body shrinkage. Cells were elongated and fragmented while nuclei were condensed or fragmented.

**Fig 10:**
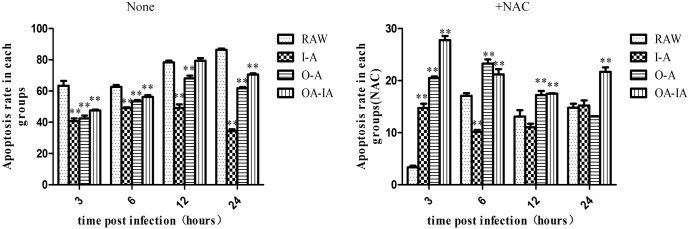
Cell apoptotic rate of different treatment groups. Cells were pretreated by either vehicle control or NAC, followed by *Brucella melitensis* 16M infection. At 3, 6, 12, and 24 h after infection, cells were digested and collected, followed by flow cytometry detection in accordance with the apoptosis kit instructions.

Transmission electron microscopy images of apoptotic cells are shown in S8 Fig. Compared with the control group, cell apoptosis in the experimental group was more significant. Control cells became smaller in size and the cytoplasm appeared dense. Chromatin underwent condensation into compact patches in the nucleus and the cell membrane began blebbing. However, apoptosis in the experimental group was more severe. Chromatins in the apoptotic cells were highly condensed and marginalized, with characteristics of late apoptosis. Nuclei broke apart into apoptotic bodies. After NAC pretreatment, cell apoptosis and apoptotic cell numbers were reduced with only a small fraction of cells showing nuclei degradation. Flow cytometry results showed apoptotic rates in different treatment groups (S9 Fig and Fig 10). With the elongation of the *B*. *melitensis* 16M infection time, the apoptosis rate was also increased. NAC pretreatment significantly reduced the apoptotic rate, indicating that *B*. *melitensis* 16M infection in macrophages induced apoptosis via the ROS pathway.

### Caspase-3 Protein Levels in *B*. *melitensis* 16M-Infected Cells

Western blot was performed to detect the expression levels of Caspase-3 protein. ImageJ software (NIH, USA) was used to analyze Caspase-3 abundance relative to the internal control. The results showed that Caspase-3 protein expression levels in the experimental group were significantly lower than that in the control group (P < 0.01). After NAC pretreatment, Caspase-3 protein expression levels in the experimental groups were generally decreased, but were still higher than the in control group (S10 Fig), which suggested that *B*. *melitensis* 16M induced apoptosis of RAW264.7 cells via the ROS pathway.

## Discussion

In the innate immune cell cytoplasma there is a class of multi-protein complexes called inflammation complexes, which can not only quickly identify various intracellular pathogenic microorganisms and their products, but also serves as a sensor for the host metabolic stress stimuli[27]. These inflammation complexes activate Caspase-1, affect the maturation and secretion of certain cytokines, regulate the immune response and inflammation, and play a critical role in innate immunity defense against pathogens[28, 29]. These inflammation complexes can be activated by multiple stimulants[30]. In this experiment, a macrophage RAW264.7 model was used to observe the effects of *B*. *melitensis* 16M on the activation of inflammatory complexes and the role of ROS therein. *Brucella* induced ROS production in RAW264.7 cells in a time-dependent manner. Overexpression or rescue of AIR also promoted ROS production. Caspase-1 could activate NLRP3 and the AIM2 inflammasome, thereby causing inflammation. Caspase-1 was consistent with the changes of ASC, which also confirmed that *Brucella* infection, similar with other intracellular parasites, activated the NLRP3 inflammasome and shared the same molecular mechanisms with a variety of other pathogenic microorganisms, especially facultative intracellular parasites. After the AIR gene was silenced, *Brucella*-induced atypical autophagy was further increased, demonstrating that the AIR genes were not only involved in inflammation, but also in autophagy.

Excess ROS can alter the activity of a particular enzyme by redox reaction, and participate in the regulation of autophagy and programmed cell death, and thus, exert adverse effects on the body[31]. We found that after *B*. *melitensis* 16M infection, GFP-LC3 was dispersed in the control cells while experimental groups showed punctate aggregates, which indicated cell autophagy upon the infection of *B*. *melitensis* 16M. Mitochondria are important intracellular energy production organelles, and the major source of ROS. The structure, function, and dynamics of mitochondria and ROS from mitochondria are closely related with the autophagic process[32]. We confirmed that after AIR interference, GFP-LC3 punctate aggregates became more significant, indicating that the interference of AIR promoted autophagy. Furthermore, we found that NAC pretreatment decreased intracellular GFP-LC3 punctate aggregates, suggesting that autophagy was inhibited. I-A and O-A cells showed less abnormal mitochondrial aggregates than the control cells, suggesting that NAC could reduce mitochondrial damage caused by *B*. *melitensis* 16M. If the body is dysfunctional, it may cause mitochondrial dysfunction and excessive ROS can damage the mitochondria itself and other cellular components[33].

Studies have shown that after cells undergo apoptosis, ROS production is increased, and the increase of ROS could promote cell apoptosis. Compared with the control group, *B*. *melitensis* 16M infection resulted in higher cell apoptosis, suggesting that AIR domain were involved in cell apoptosis. The dynamics of ROS determines the different reactions of cells in response to the same signal and inherent ROS levels can determine the sensitivity of certain cells to a particular signal[34]. In our current findings, the ratio of Bax/Bcl-2 in the I-A, O-A, and OA-IA groups was significantly higher than in the control group, indicating that Bcl-2 can inhibit ROS induction, which is consistent with previous research results[35]. After receiving the signals, different levels of ROS determine cell apoptosis, necrosis, or the conversion from apoptosis to necrosis[36]. Cell apoptosis was significantly reduced in *B*. *melitensis* 16M-infected and NAC-pretreated cells, indicating that there was a connection between AIR and ROS. Thus, it was substantiated that *B*. *melitensis* 16M infection-induced cell apoptosis via the ROS pathway.

## Conclusions

In conclusion, *B*. *melitensis* 16M is capable of regulating the effects of the AIR domain on inflammatory factors, autophagy, and apoptosis in mouse macrophage via the ROS signaling pathway. The AIR domain participates in the inflammatory response and activation of NLRP3 promoted by *B*. *melitensis* 16M through the ROS signaling pathway. After the interference of the AIR domain, *B*. *melitensis* 16M-induced atypical autophagy was promoted, indicating that the AIR domain is not only involved in the cellular inflammatory response, but also in the autophagy. In addition, *B*. *melitensis* 16M promotes apoptosis, which was positively correlated with the time of infection. The apoptotic rate of the NAC-pretreatment group was significantly lower than that of the untreated group, which showed that AIR could affect *B*. *melitensis* 16M apoptosis induced by *B*. *melitensis* 16M via ROS pathway. The study provides a reliable theoretical basis for the systematic exposition of the molecular mechanism of *B*. *melitensis* 16M-persistent infection.

## Supporting Information

S1 Fig RAW264.7 cells were transfected with recombinant lentivirus. RAW264.7 cells were seeded into 6-well plates and infected with 3–5 mL lentivirus. Polybrene (1 mg/mL) was added to the cell medium at a final concentration of 1 μg/mL, and was mixed well, and then placed in a 37°C, 5% CO_2_ incubator. After 12 h, the cell morphology was observed and infected with a second round of lentivirus (or control medium) and polybrene. Cells were placed back in a 37°C, 5% CO_2_ incubator. Cell fluorescence was observed 48 h later.
(TIF)

S2 Fig DCFH-DA probe was used to detect *B*. *Melitensis* 16M-induced ROS production levels. The interference groups of I-A cells, overexpression group of O-A cells, overexpression-interference group of OA-IA cells, and the normal group of RAW264.7 cells were seeded into a 35-mm confocal dish. At 6 and 12 h after infection, the DCFH-DA probe was added into the cells to sufficiently cover the cells and they were incubated at 37°C for 20 min, followed by 4% paraformaldehyde solution incubation for 20–30 min. After the PFA was removed, 600 μL of PBS was added. Confocal laser microscope was used to detect ROS production.
(TIF)

S3 Fig Mito-ID^®^ Red Dye was used to detect *B*. *Melitensis* 16M-induced mitochondrial distribution. The interference group of I-A cells, overexpression group of O-A cells, overexpression-interference group of OA-IA cells, and the normal group of RAW264.7 cells were seeded into a 35 mm confocal dish. At 6 and 12 h after infection, MIito-ID^®^ Red was added to stain the cells for 15–30 min. Confocal laser microscope was used to detect mitochondria distribution.
(TIF)

S4 Fig Transmission electron microscope was used to observe the distribution of mitochondria in each group of cells. Both the untreated and NAC-pretreated groups were infected with *B*. *Melitensis* 16M. At 6 and 12 h after infection, cells were digested with 0.25% trypsin. After trypsin was discarded, cells were fixed with 4% glutaraldehyde. Cells were fixed again with 1% osmium tetroxide, followed by ethanol dehydration and penetration of the epoxy resin. Samples were sectioned with microtome and stained with uranyl acetate and lead citrate. Mitochondria were observed under a transmission electron microscope.
(TIF)

S5 Fig NLRP3 and Caspase-1 expression levels were detected by Western blot. Both the untreated and NAC-pretreated groups were infected with *B*. *Melitensis* 16M. At 0, 3, 6, 12, and 24 h after infection, the cells were lysed by RIPA buffer on ice for 5–10 min. The lysate was collected and subjected to Western blot detection.
(TIF)

S6 Fig Distribution of autophagosomes were examined under transmission electron microscope. Both the untreated and NAC-pretreated groups were infected with *B*. *Melitensis* 16M. At 6 and 12 h after infection, the cells were digested with 0.25% trypsin. After trypsin was discarded, cells were fixed with 4% glutaraldehyde. Cells were fixed again with 1% osmium tetroxide, followed by ethanol dehydration, and penetration of the epoxy resin. Samples were sectioned with microtome, and stained with uranyl acetate and lead citrate. Autophagosome were observed under a transmission electron microscope. (A, a) electron microscope of *B*. *Melitensis* 16M, (B, b) NC group, (C, c) I-A group, (D, d) O-A group, and (E, e) OA-IA group.
(TIF)

S7 Fig Western blot to detect the expression of p62 protein. Both the untreated and NAC-pretreated groups were set up and infected with *B*. *Melitensis* 16M. At 0, 3, 6, 12, and 24 h after infection, the cells were placed on ice and lysed by RIPA buffer for 5–10 min. The lysate was collected and subjected to Western blot detection.
(TIF)

S8 Fig TEM to detect cell apoptosis in each group. Both the untreated and NAC-pretreated groups were infected with *B*. *Melitensis* 16M. At 6 h after infection, the cells were digested with 0.25% trypsin. After the trypsin was discarded, the cells were fixed with 4% glutaraldehyde. Cells were fixed again with 1% osmium tetroxide, followed by ethanol dehydration, and penetration of the epoxy resin. Samples were sectioned with a microtome, and stained with uranyl acetate and lead citrate. Apoptotic bodies were observed under a transmission electron microscope.
(TIF)

S9 Fig Flow cytometry to detect cell apoptotic rate of different treatment groups. Cells were pretreated by either vehicle control or NAC, followed by *B*. *Melitensis* 16M infection. At 3, 6, 12, and 24 h after infection, cells were digested and collected, followed by flow cytometry detection in accordance with the apoptosis kit instructions.
(TIF)

S10 Fig Cells were pretreated by either vehicle control or NAC followed by *B*. *Melitensis* 16M infection. In 3, 6, 12, and 24 h after infection, the cell lysate was collected and detected by Western blot.
(TIF)
