# Supplementary figures and images for: Brucella Melitensis 16M Regulates the Effect of AIR Domain on Inflammatory Factors, Autophagy, and Apoptosis in Mouse Macrophage through the ROS Signaling Pathway
Source: PLoS One. 2016 Dec 1;11(12):e0167486. doi: 10.1371/journal.pone.0167486 (PMC5132199; doi:10.1371/journal.pone.0167486)

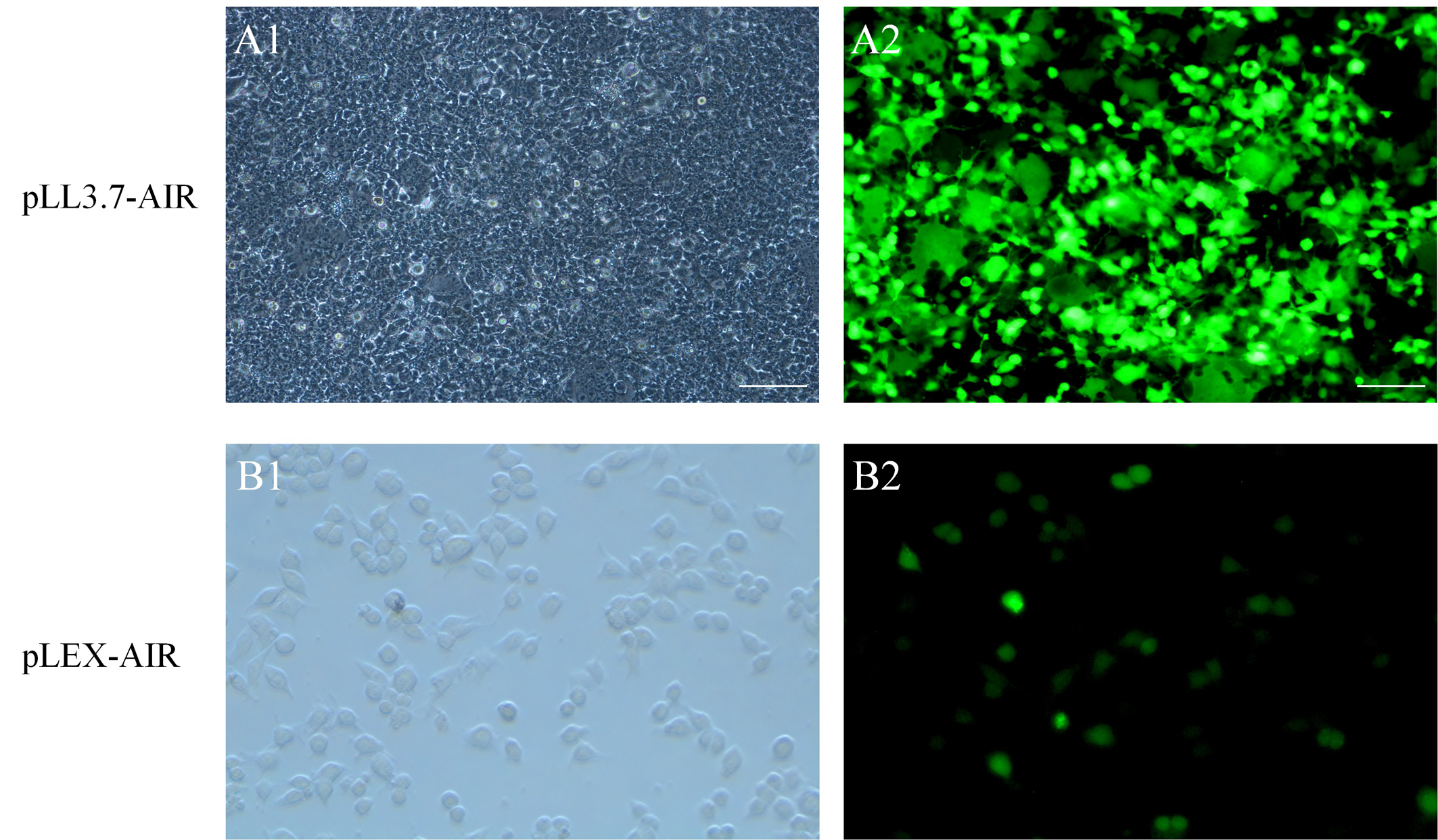

Supplement: S1 Fig — RAW264.7 cells were seeded into 6-well plates and infected with 3–5 mL lentivirus. Polybrene (1 mg/mL) was added to the cell medium at a final concentration of 1 μg/mL, and was mixed well, and then placed in a 37°C, 5% CO2 incubator. After 12 h, the cell morphology was observed and infected with a second round of lentivirus (or control medium) and polybrene. Cells were placed back in a 37°C, 5% CO2 incubator. Cell fluorescence was observed 48 h later. (TIF) [file pone.0167486.s001.tif]

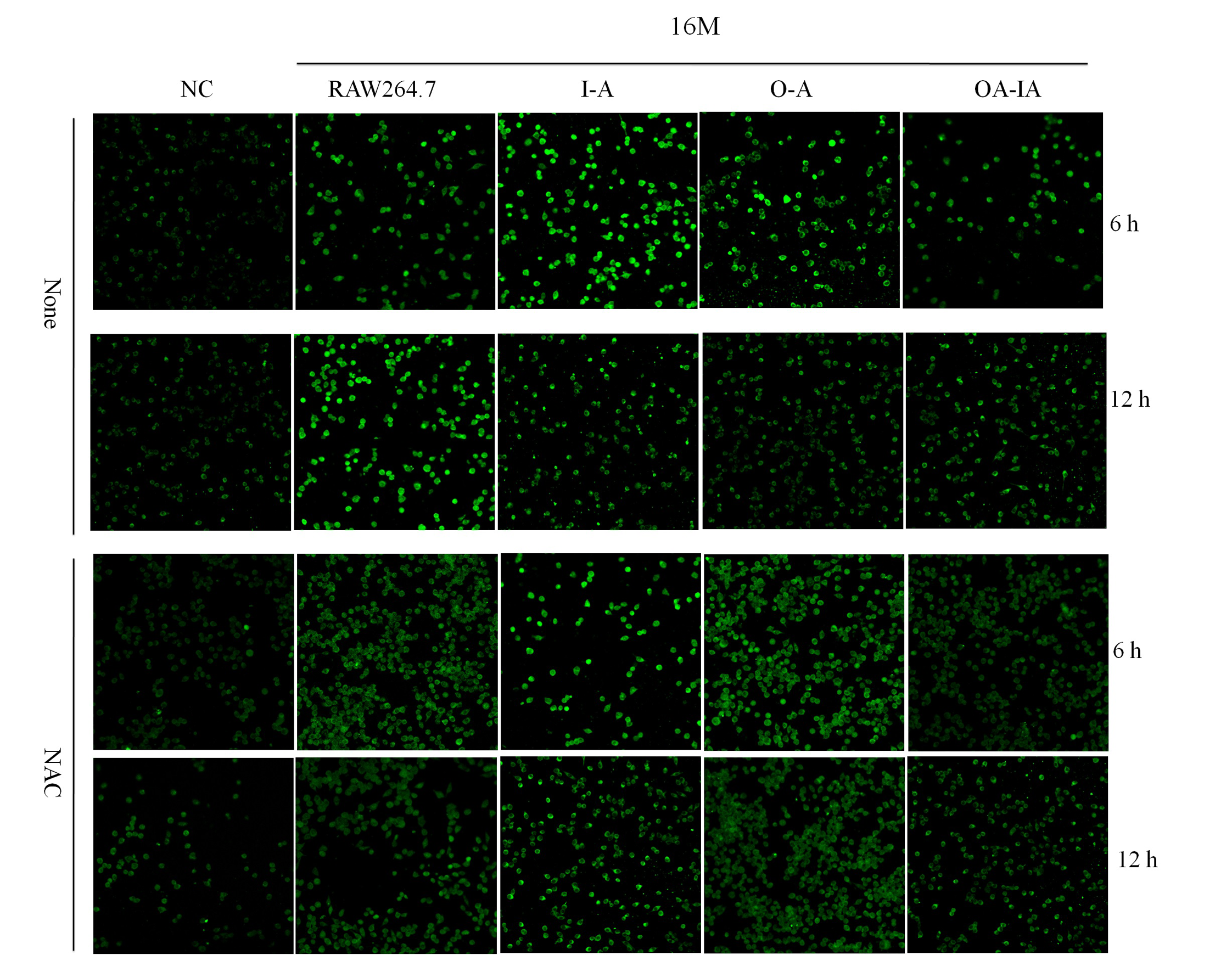

Supplement: S2 Fig — The interference groups of I-A cells, overexpression group of O-A cells, overexpression-interference group of OA-IA cells, and the normal group of RAW264.7 cells were seeded into a 35-mm confocal dish. At 6 and 12 h after infection, the DCFH-DA probe was added into the cells to sufficiently cover the cells and they were incubated at 37°C for 20 min, followed by 4% paraformaldehyde solution incubation for 20–30 min. After the PFA was removed, 600 μL of PBS was added. Confocal laser microscope was used to detect ROS production. (TIF) [file pone.0167486.s002.tif]

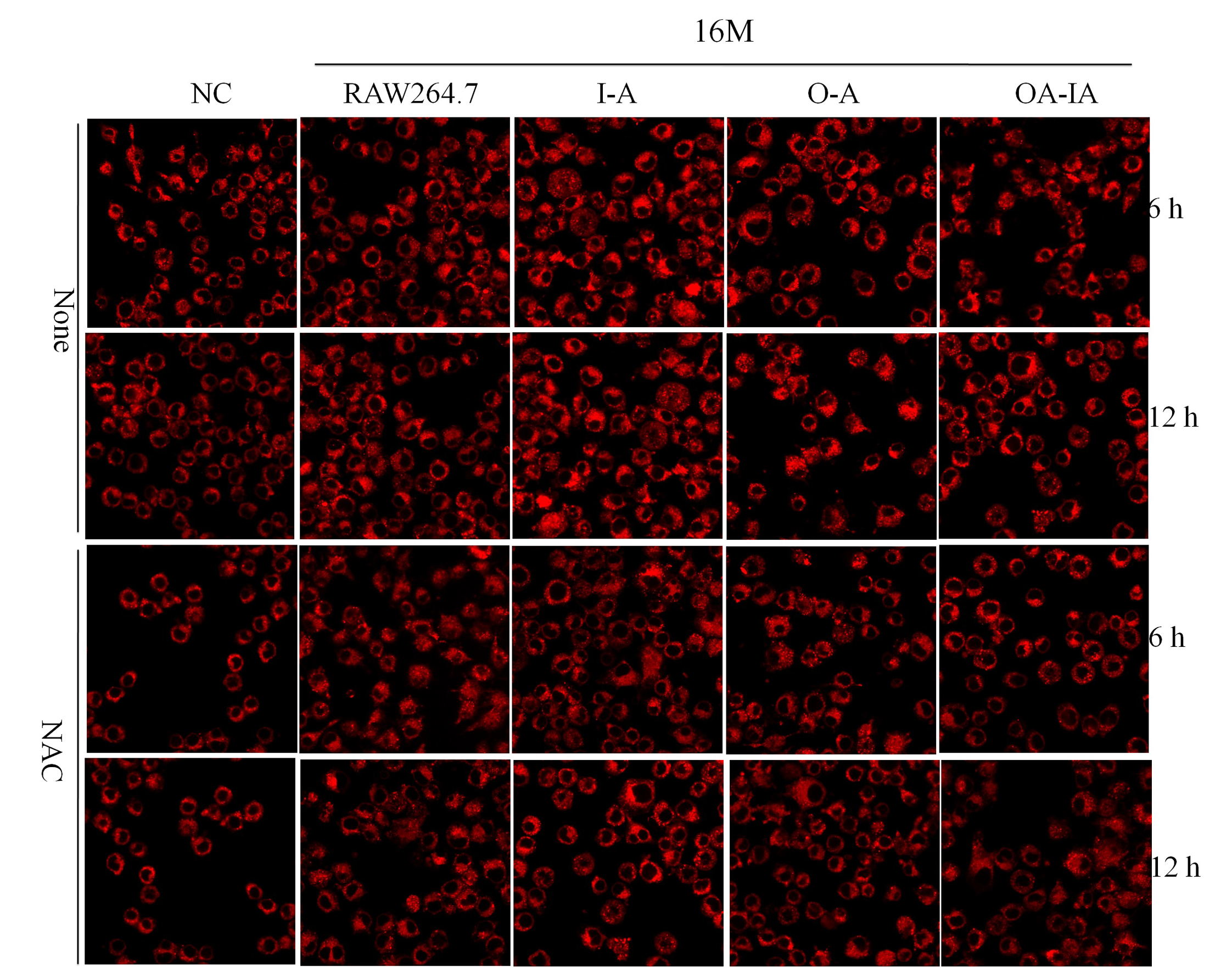

Supplement: S3 Fig — The interference group of I-A cells, overexpression group of O-A cells, overexpression-interference group of OA-IA cells, and the normal group of RAW264.7 cells were seeded into a 35 mm confocal dish. At 6 and 12 h after infection, MIito-ID® Red was added to stain the cells for 15–30 min. Confocal laser microscope was used to detect mitochondria distribution. (TIF) [file pone.0167486.s003.tif]

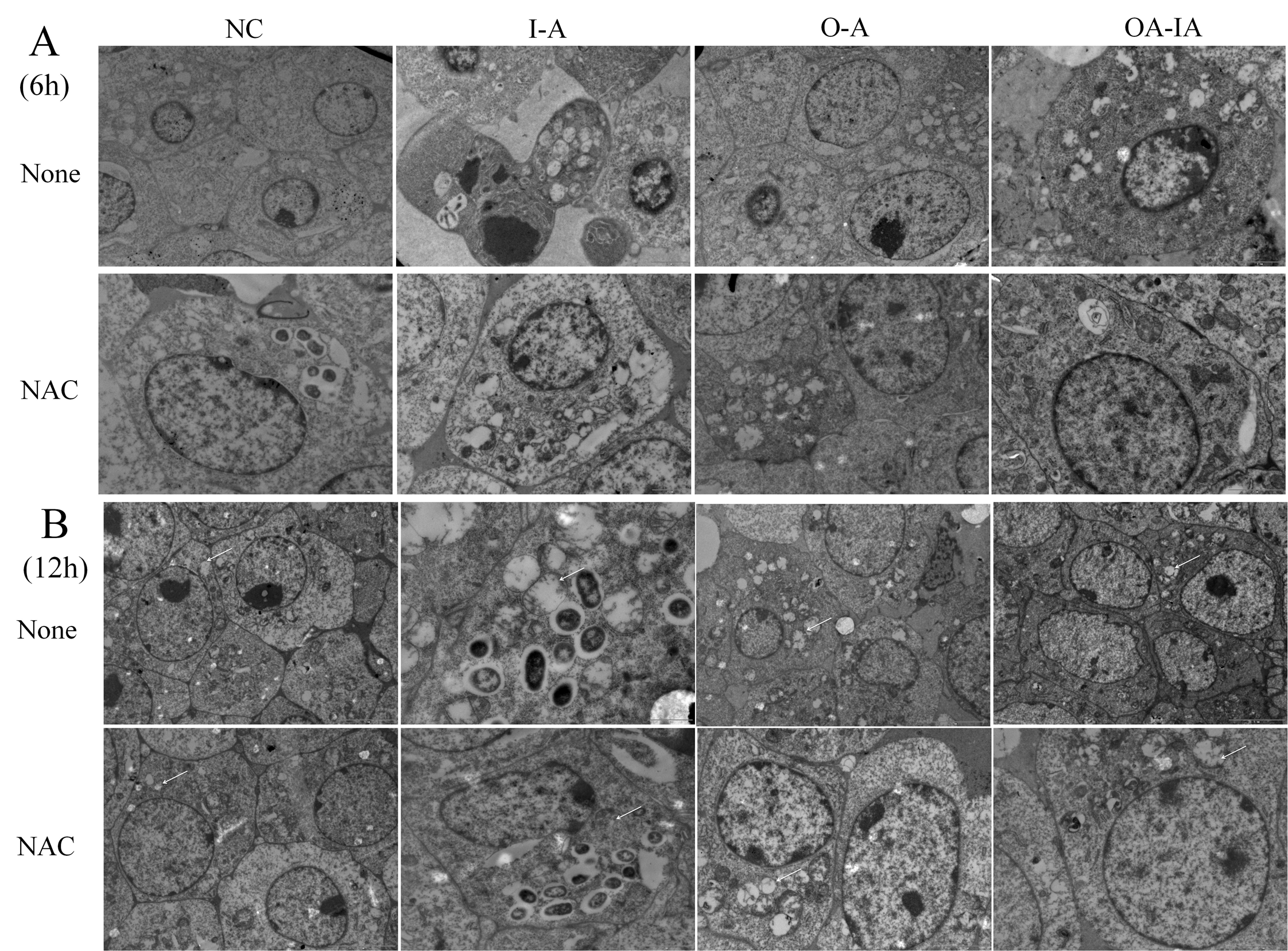

Supplement: S4 Fig — Both the untreated and NAC-pretreated groups were infected with B. Melitensis 16M. At 6 and 12 h after infection, cells were digested with 0.25% trypsin. After trypsin was discarded, cells were fixed with 4% glutaraldehyde. Cells were fixed again with 1% osmium tetroxide, followed by ethanol dehydration and penetration of the epoxy resin. Samples were sectioned with microtome and stained with uranyl acetate and lead citrate. Mitochondria were observed under a transmission electron microscope. (TIF) [file pone.0167486.s004.tif]

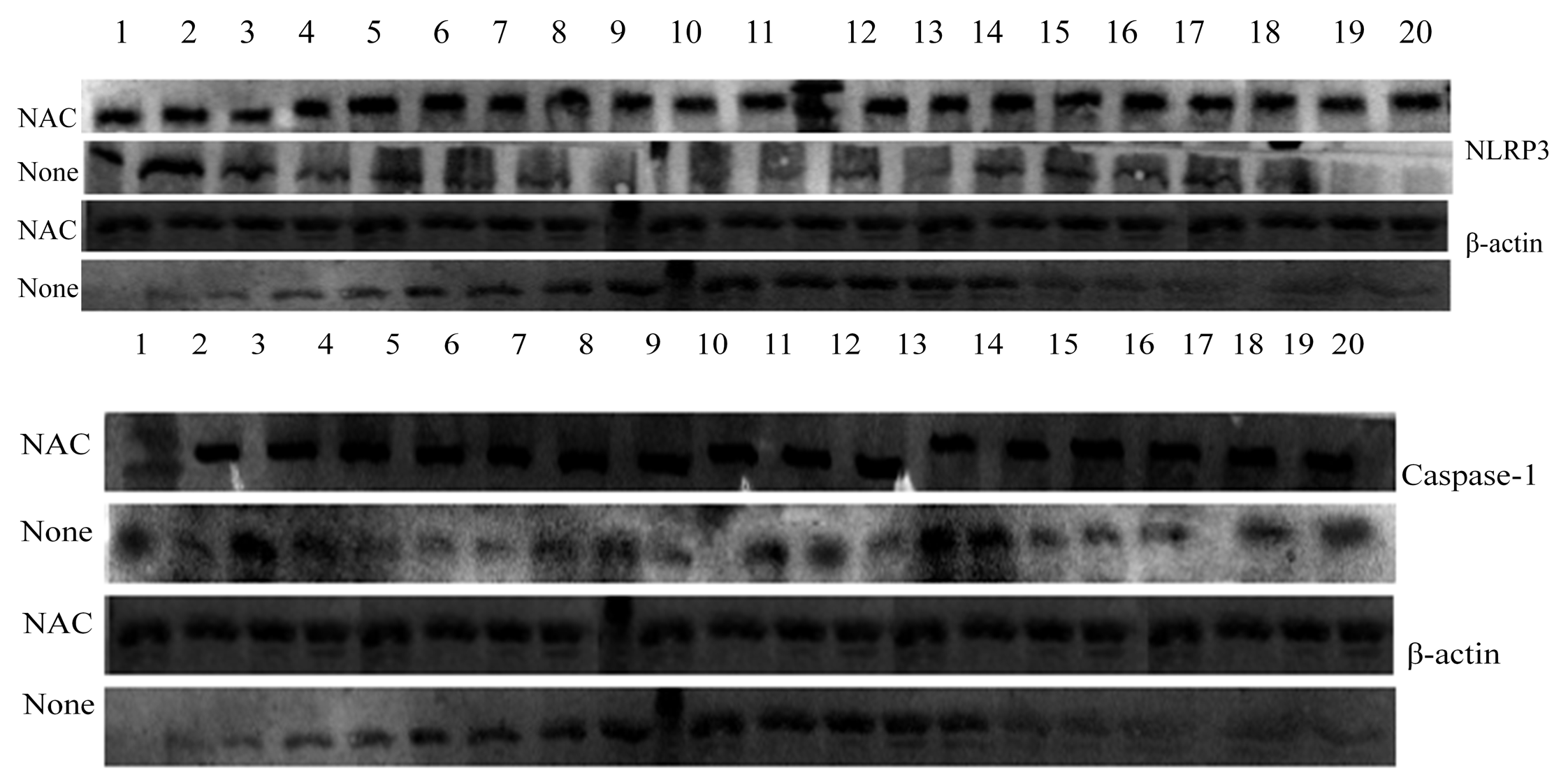

Supplement: S5 Fig — Both the untreated and NAC-pretreated groups were infected with B. Melitensis 16M. At 0, 3, 6, 12, and 24 h after infection, the cells were lysed by RIPA buffer on ice for 5–10 min. The lysate was collected and subjected to Western blot detection. (TIF) [file pone.0167486.s005.tif]

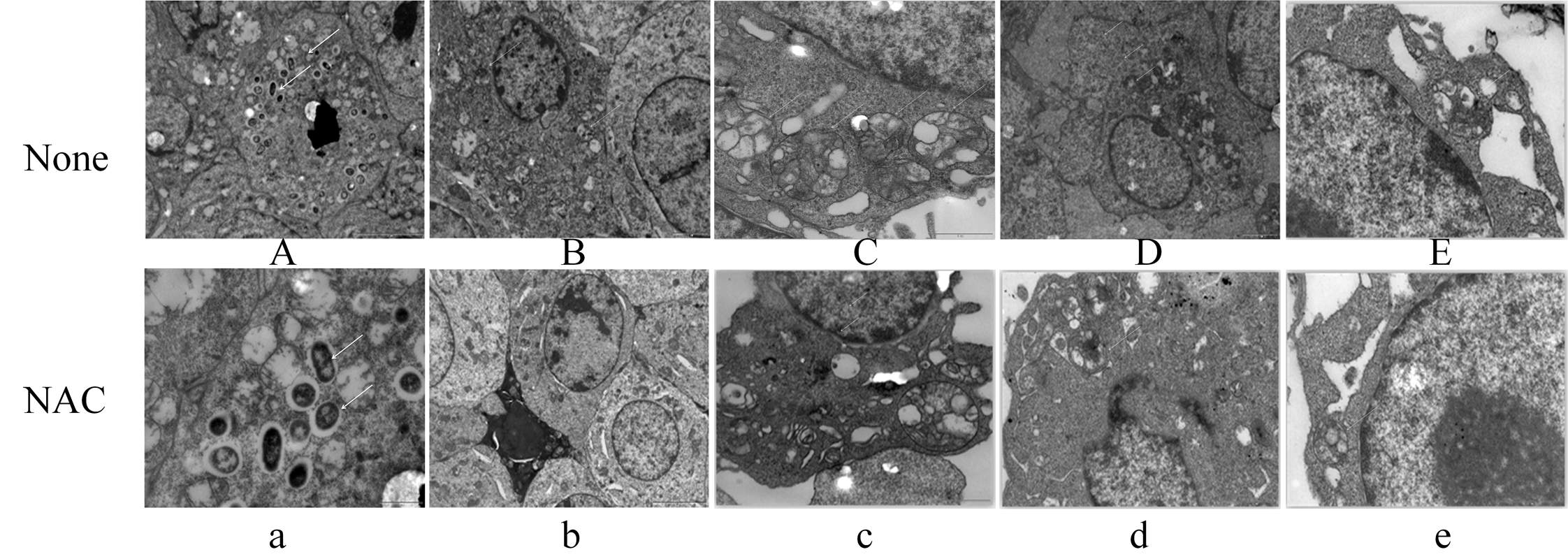

Supplement: S6 Fig — Both the untreated and NAC-pretreated groups were infected with B. Melitensis 16M. At 6 and 12 h after infection, the cells were digested with 0.25% trypsin. After trypsin was discarded, cells were fixed with 4% glutaraldehyde. Cells were fixed again with 1% osmium tetroxide, followed by ethanol dehydration, and penetration of the epoxy resin. Samples were sectioned with microtome, and stained with uranyl acetate and lead citrate. Autophagosome were observed under a transmission electron microscope. (A, a) electron microscope of B. Melitensis 16M, (B, b) NC group, (C, c) I-A group, (D, d) O-A group, and (E, e) OA-IA group. (TIF) [file pone.0167486.s006.tif]

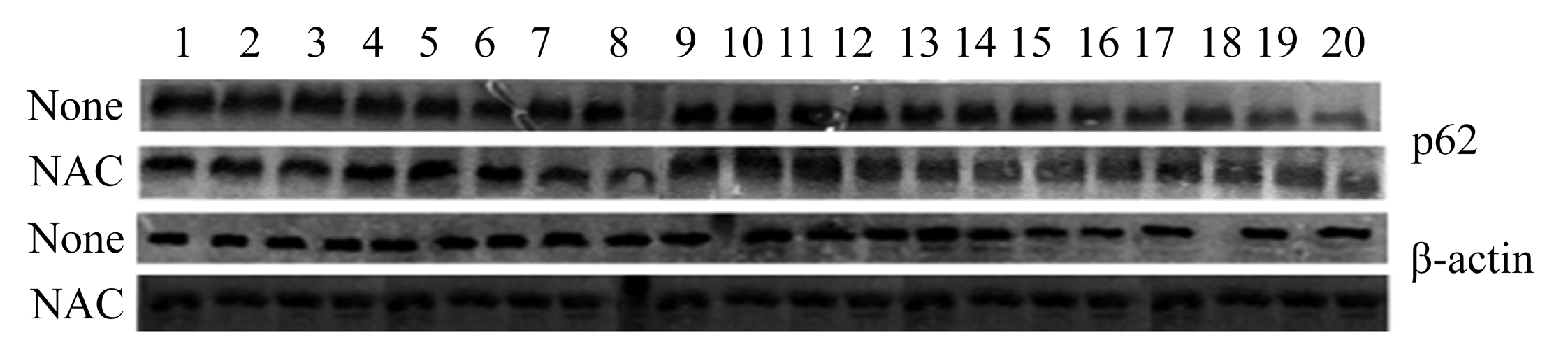

Supplement: S7 Fig — Both the untreated and NAC-pretreated groups were set up and infected with B. Melitensis 16M. At 0, 3, 6, 12, and 24 h after infection, the cells were placed on ice and lysed by RIPA buffer for 5–10 min. The lysate was collected and subjected to Western blot detection. (TIF) [file pone.0167486.s007.tif]

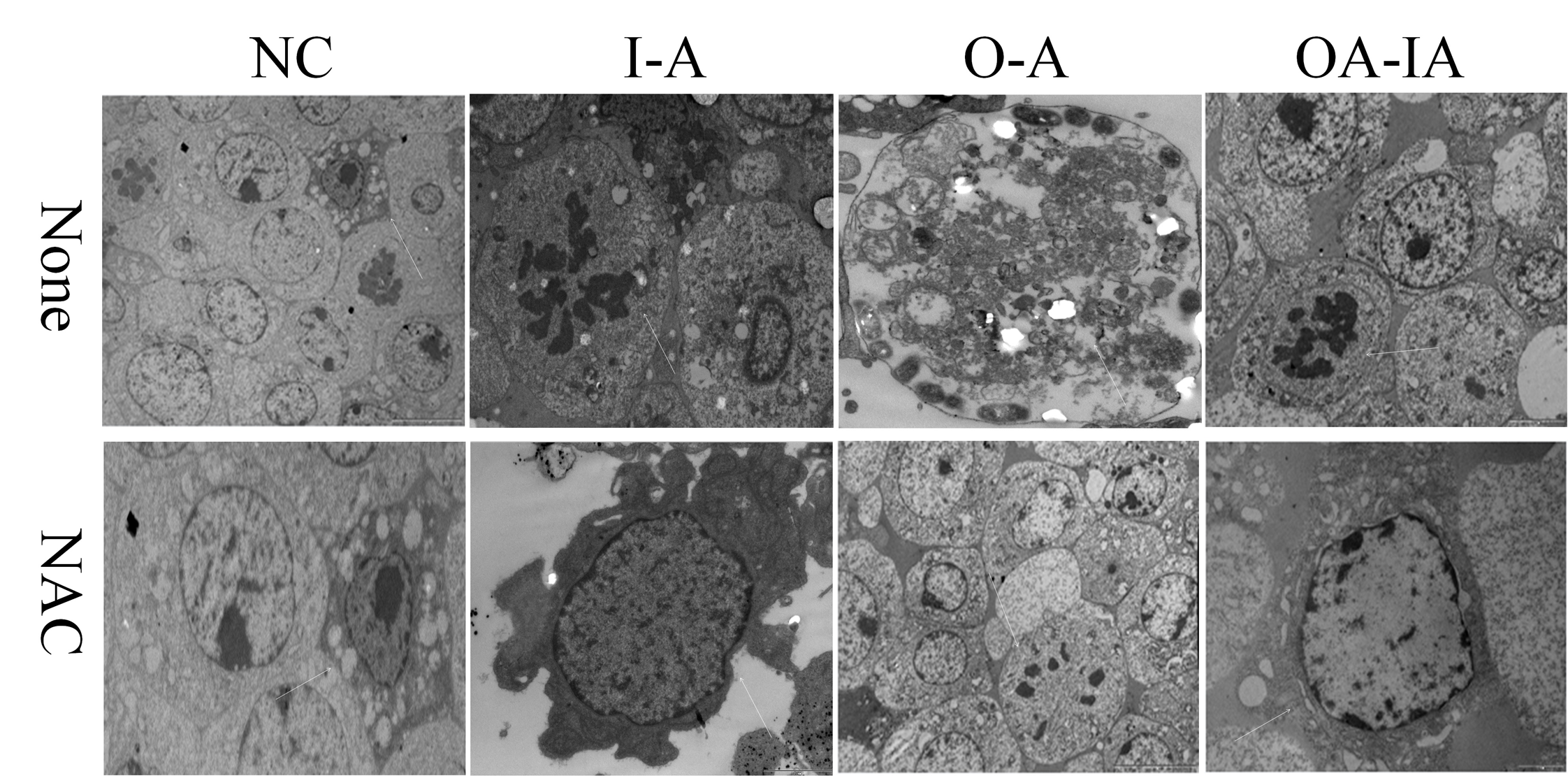

Supplement: S8 Fig — Both the untreated and NAC-pretreated groups were infected with B. Melitensis 16M. At 6 h after infection, the cells were digested with 0.25% trypsin. After the trypsin was discarded, the cells were fixed with 4% glutaraldehyde. Cells were fixed again with 1% osmium tetroxide, followed by ethanol dehydration, and penetration of the epoxy resin. Samples were sectioned with a microtome, and stained with uranyl acetate and lead citrate. Apoptotic bodies were observed under a transmission electron microscope. (TIF) [file pone.0167486.s008.tif]

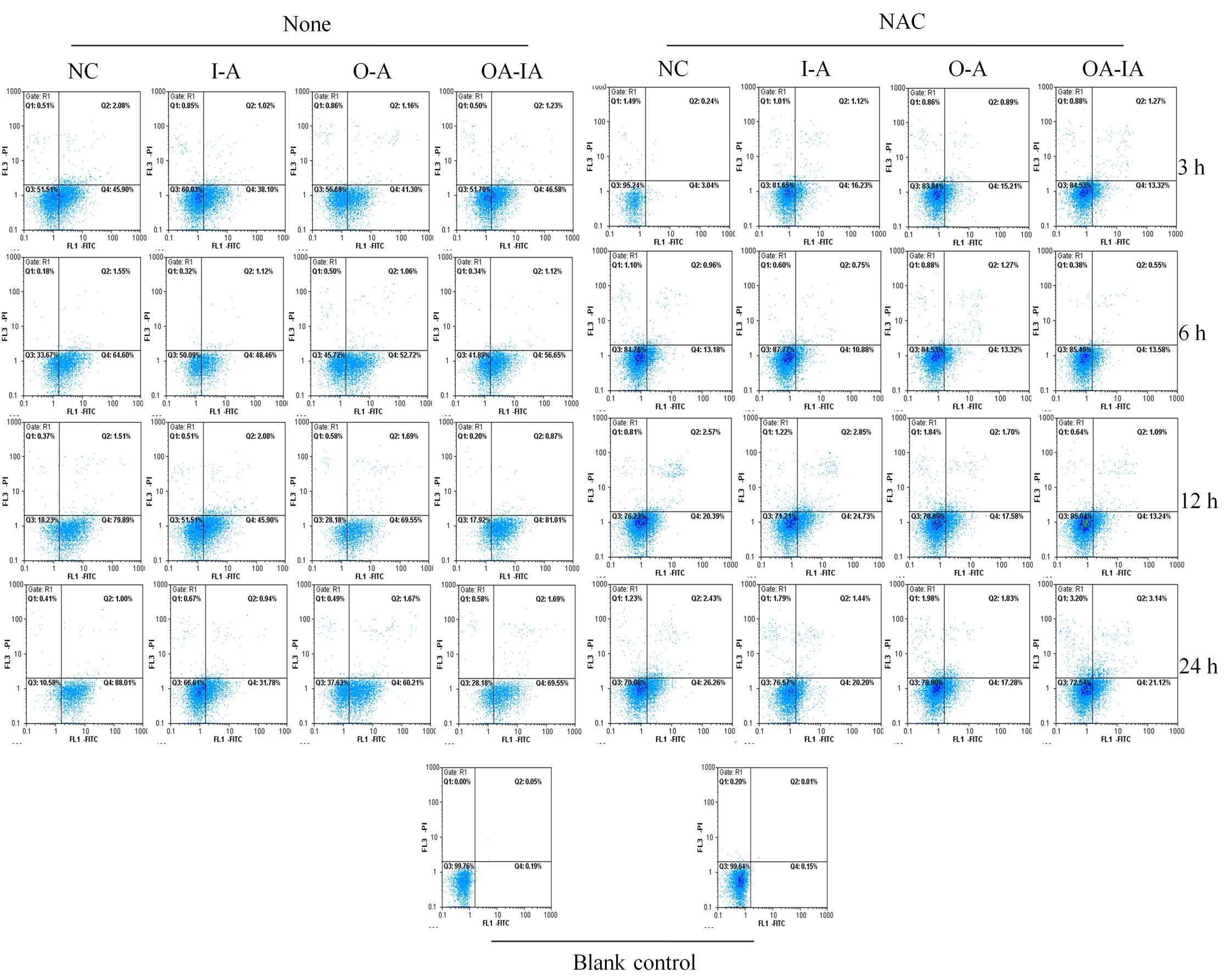

Supplement: S9 Fig — Cells were pretreated by either vehicle control or NAC, followed by B. Melitensis 16M infection. At 3, 6, 12, and 24 h after infection, cells were digested and collected, followed by flow cytometry detection in accordance with the apoptosis kit instructions. (TIF) [file pone.0167486.s009.tif]

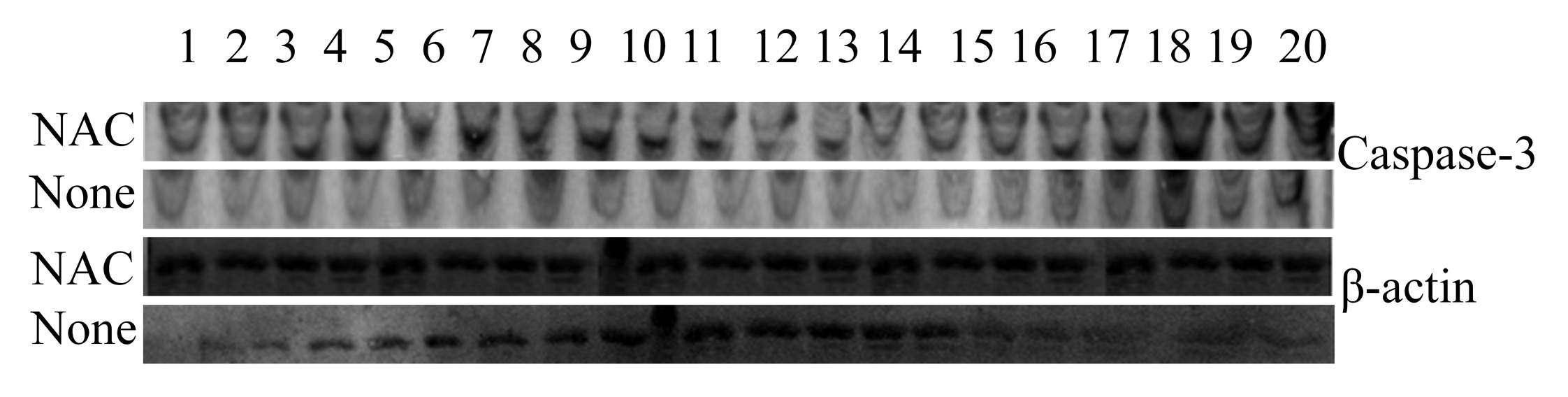

Supplement: S10 Fig — In 3, 6, 12, and 24 h after infection, the cell lysate was collected and detected by Western blot. (TIF) [file pone.0167486.s010.tif]
